# Deficiency Citations in Nursing Homes That Predominantly Serve Residents With Serious Mental Illness

**DOI:** 10.1093/geroni/igaa057.292

**Published:** 2020-12-16

**Authors:** Dylan Jester, Kathryn Hyer, John Bowblis

**Affiliations:** 1 University of South Florida, Tampa, Florida, United States; 2 Miami University, Oxford, Ohio, United States

## Abstract

Studies suggest that nursing homes (NHs) that predominantly serve residents with serious mental illness (SMI) are of worse quality due to poor resources (i.e., high Medicaid-paying census) and lower staffing. We used national Certification and Survey Provider Enhanced Reports (CASPER) data to examine the deficiencies issued to NHs from 37,800 recertification inspections of 14,582 unique NHs from 2014 to 2017. NHs were categorized into “low-SMI” and “high-SMI” facilities using the lowest and highest quartiles, respectively, of the proportion of residents in the NH with SMI. Bivariate analyses were used to assess for differences between low-SMI and high-SMI NHs in the number of deficiencies, the deficiency score (a point-based metric developed by the Centers for Medicare & Medicaid Services), and the scope and severity of deficiencies. In total, there were 245,178 deficiencies issued. In comparison to low-SMI NHs, high-SMI NHs received a greater deficiency score and more deficiencies per survey (p<.001). Deficiencies given to high-SMI NHs were associated with greater risk of harm (p<.001) and were of wider scope (p<.001). High-SMI NHs were cited 215% more often for resident abuse or neglect and 61% more often for the policies that prohibit and monitor for risk of abuse and neglect in comparison to low-SMI NHs. In conclusion, high-SMI NHs were documented for providing worse care to residents, with one particular area of concern being the heightened risk of resident abuse and neglect. Implications for policy and practice will be discussed.

